# Preparing for Delivery of Patient Falls Prevention Education in Hospitals: The Consumer Perspective

**DOI:** 10.1111/hex.70878

**Published:** 2026-09-21

**Authors:** Hazel Heng, Jacqueline Francis‐Coad, Caroline Bulsara, Cheng Yen Loo, Meg E. Morris, Adam Semciw, Catherine M. Said, Stephen Peterson, Anne‐Marie Hill

**Affiliations:** ^1^ Allied Health Northern Health, Melbourne Victoria Australia; ^2^ School of Allied Health, Human Services and Sport, La Trobe University, Bundoora Victoria Australia; ^3^ School of Health and Clinical Sciences, The University of Western Australia Crawley WA Australia; ^4^ Western Australian Centre for Health & Ageing, University of Western Australia Perth WA Australia; ^5^ Institute Health Research, Faculty of Medicine, Nursing and Midwifery & Health Sciences, The University of Notre Dame Australia Fremantle WA Australia; ^6^ ARCH, La Trobe University, Bundoora Victoria Australia; ^7^ The Victorian Rehabilitation Centre, Glen Waverley Victoria Australia; ^8^ Physiotherapy Department Western Health, St Albans Victoria Australia; ^9^ Physiotherapy, Melbourne School of Health Sciences, The University of Melbourne, Parkville Victoria Australia; ^10^ Australian Institute for Musculoskeletal Science (AIMSS) Australia; ^11^ Royal Perth Hospital Research Foundation, Royal Perth Hospital Perth WA Australia

**Keywords:** accidental falls, consumer engagement, implementation, older adults, patient education, prevention, safety in hospitals

## Abstract

**Background:**

Falls in hospital are a serious risk to older patients' recovery. Patient education programmes that provide falls prevention advice are recommended. Garnering the perspectives of older adults about implementing such programmes could improve how health professionals engage with hospital patients to educate them and increase patient enactment of falls prevention advice.

**Methods:**

Two forums, in two states of Australia, were conducted using a modified World Café methodology. Older consumers who had previous admissions to hospital shared their perspectives on three conversation topics: (i) delivery of a purpose‐designed patient falls prevention education programme called Safe Recovery; (ii) ward‐level support for programme delivery; and (iii) organisational‐level support for the programme. Table notes were combined and analysed using inductive content analysis.

**Results:**

Older consumers who could communicate in English from Western Australia (n = 17) and Victoria (n = 16) participated in the forums. Their perspectives appeared to concur that implementation of the Safe Recovery Programme for falls prevention on hospital wards requires staff attention to the right timing for patients, personalisation of the education and multiple modes of delivery to optimise programme exposure. Consumers advised that ward‐level support to remember and enact suitable strategies should be provided by staff, families, hospital volunteers and peers. They requested clear communication between staff that ensured they repeatedly and consistently reinforced patient participation and provided progress reports to patients on falls prevention. Organisations can support implementation by taking ownership for ensuring patients receive falls prevention education, endorse staff falls education training and allocate funding for programme sustainability. The group consensus was everyone can work together to prioritise falls prevention in hospitals.

**Conclusion:**

Older consumers expressed their enthusiasm to partner in planning how a patient falls prevention education programme is delivered on hospital wards. Consumer engagement can provide valuable stakeholder feedback about processes and resources required for delivery of patient falls prevention education, which may lead to successful delivery by hospitals.

**Patient or Public Contribution:**

The research team includes a consumer investigator (SP) who has provided feedback and input at all stages of the research, including project design, study procedures, data analysis and manuscript review. The education programme's original design and development was centred around patient and public involvement. The participants in this study who attended the world café forums were older people with lived experience of hospital admissions and caregivers of older people admitted to hospital.

## 1 Introduction

Falls occurring in hospitals continue to be a significant issue globally [1, 2] particularly for older adults or those with frailty or multi‐morbidity [3]. The rate of hospital falls can range from 3 to 18 falls per 1000 bed days [4, 5, 6] depending on the setting (acute, rehabilitation or specialty wards such as psychogeriatric wards). Falls in hospital can lead to serious consequences for patients such as minor or severe injuries [1, 7] significant psychological impacts [8] and institutionalisation or mortality [9]. Despite this, most older adults do not believe they are at risk of falls whilst in hospital so improving self‐perception of risk and knowledge about how to prevent falls is needed [10, 11]. Patient education has been most effective in reducing hospital falls rates [1, 5, 12] and is a key recommendation of the world guidelines for falls prevention and management for older adults [13]. Despite this, consumer‐led research has shown that within the context of hospitalisation, older adults were unsure of how or when to take action to mitigate falls, and if they did receive falls education it was viewed as inadequate, generic and inconsistent [14].

The design, delivery and quality of patient falls prevention education programmes can impact patient outcomes. Improvements in patient knowledge and awareness to reduce falls when in hospital have been associated with well‐designed education programmes [15]. However, a systematic review of hospital clinical practice guidelines revealed that not all guidelines included falls prevention education programmes, and most provided limited guidance on how to deliver education programmes to patients [2]. The Safe Recovery Programme (SRP) is an evidence‐based patient education programme for falls prevention in hospital settings where a health professional delivers education to the patient at the bedside via verbal communication, a short video and a booklet [5]. It is grounded in health behaviour change, adult learning principles and uses tailored multi‐media resources for delivery. It has been shown to increase patient capability, create opportunities and encourage motivation to support behaviour change and reduce falls and injurious falls rates [5, 16]. Whilst the revised SRP and resources have been co‐designed with stakeholders, including consumers and healthcare providers [17], it is important to continue the co‐design process with consumers regarding future implementation. Thus, engaging consumers to understand their perspectives on how the SRP should be delivered will be key in facilitating effective delivery.

To achieve real‐world change, interventions must be successfully implemented; hence, it is important to ensure that the intervention is contextually appropriate and responsive to the specific needs of consumers and their relevant settings [18, 19, 20]. In the context of older patients on hospital wards, involving older consumers with lived experience of hospital admissions can provide a valuable perspective for planning effective programme implementation such as the SRP [19, 20]. Such engagement can also assist in identifying priorities and addressing gaps prior to implementation, thus reducing the risk of implementation failure [18, 20]. Therefore, the objective of this study was to explore consumer perspectives around processes and resources to facilitate the successful delivery of the revised SRP in hospitals.

## 2 Methods

### 2.1 Design

Two community forums were conducted in Australia using a modified World Café methodology. This method uses an iterative process that fosters interactive conversations allowing multiple diverse perspectives to be shared and integrated [21]. The use of an informal, café style atmosphere better encourages participants (older adult consumers) to have a voice about issues of importance to them and enables them to become co‐creators with the researchers in planning [22] implementation of hospital falls prevention education. Ethics approval (ID: 2024/ET000566) has been provided through the University of Western Australia and La Trobe University Human Research Ethics Committees for Western Australia and Victoria, respectively.

### 2.2 Participants and Setting

A purposeful sampling method was utilised. Participants were eligible if they were (i) older adults (aged 65 years and above) who had been admitted to an Australian hospital in the last 5 years; (ii) individuals who had previously or were currently caring for an older person who was admitted to hospital in the last 5 years; and/or (iii) consumer advocate representatives who provided care for older adults. All participants needed to have sufficient English language skills to engage in conversation in a group setting.

Both forums were held at metropolitan locations in Western Australia and Victoria to enable easy access for participants. The forums were advertised through newsletters containing contact details of a researcher, distributed throughout older peoples' organisations and flyers placed in common areas of hospitals and communal locations such as libraries and cafes. Upon contacting the researcher, a participant information sheet was provided to interested individuals to inform their decision on whether to participate.

### 2.3 Topics of Conversation

The topics of conversation within the forums were designed to explore the perspectives and insights of consumers around the effective delivery of the revised SRP to inform implementation on hospital wards.

The three topics that guided conversations during the forum were:

1. Delivery of the SRP at patient level (to determine how and when patients would prefer to have the programme delivered).
2. Ward‐level support and communication requirements (to determine what type of ward support or resources and communication processes would be needed to ensure the programme is effectively delivered to patients).
3. Organisational level support for the programme (to determine what type of organisational support is needed to support the delivery of the programme to patients).

### 2.4 Forum Procedures

The forum was conducted according to the seven core principles of the World Café: (i) creating a hospitable environment; (ii) setting the context; (iii) asking relevant questions; (iv) encouraging participation from everyone; (v) connecting diverse perspectives; (vi) listening for patterns and insights; and (vii) collecting and sharing discoveries together [21].

The lead facilitator (A‐MH) had extensive research experience, including using World Café methodology [23] and was present at both workshops. Before the commencement of the forum, the facilitators (A‐MH, MM) provided training for the table moderators and notetakers. Training included principles of the World Café methodology, the context of the revised SRP and conducting the planned forum.

The forums were set up to reflect a hospitable café‐like atmosphere with decorated tables and refreshments provided. Participants were welcomed into the forum by a research team member and invited to sit at any table in groups of five to six. All participants completed written informed consent and a demographic questionnaire.

For this study, a modified version of the world café methodology was used. The main differences were that participants remained seated at the same tables and each table discussed the same topic at the same time, rather than rotating through tables with different topics. This was considered a more pragmatic approach to cater for older adults with mobility impairments and allowing participants to become socially engaged and comfortable with their table host and fellow consumers. Table moderators remained at each table and facilitated the conversation during each topic as well as with noting down collective thoughts on large sheets of paper. Sticky notes and notepads were placed on the table for participants to use while note‐takers assisted each table in transcribing verbal responses.

The main facilitators opened each forum with a welcome address and an overview of the context and aims of the forum. The content and delivery of the SRP was explained, and SRP resources were provided for clarity. Each forum ran for approximately 2 h, with topic rounds lasting approximately 20 min. All participants were encouraged to join the conversation and write down their views and comments. At the end of each round, written materials were collected and provided to the analysts (JFC, A‐MH, CMS) for collation and summarisation. Responses to each topic were summarised and discussed briefly with the whole group before commencing the next topic of conversation. Following the third topic of conversation, a summary of responses to each topic was presented on a whiteboard for a final group discussion between participants and analysts. Forum participants were encouraged to assist in creating and confirming summaries for integration and to check responses for accuracy. Participants were provided with a voucher to thank them for their participation time.

### 2.5 Data Analysis

All transcribed open responses and written table notes from the forums, were subsequently imported into Excel spreadsheets. As little was known regarding consumers' perspectives on effectively implementing and delivering the revised SRP in hospitals, inductive content analysis was utilised [24]. Analytic rigour was ensured through a systematic and iterative coding process, maintenance of a clear audit trail, and the use of illustrative quotes to support each category. Two independent researchers (JF‐C, HH) read through the transcripts several times to familiarise themselves with the data [25]. Data from both forums were then merged to determine data saturation, and the responses were organised through category creation, and abstraction. Merging data from the two states served to increase the sample size improving representation of cultural diversity to provide a broader, more robust consumer perspective. Initially, multiple categories were derived by transferring responses onto coding sheets, followed by grouping and merging to streamline the number of categories. During the abstraction process, further input was sought from other research team members (CYL, A‐MH, SP) for assignment of content‐specific terms to each category. Similar subcategories were then grouped under a broader generic category, which ultimately led to the identification of a main category representing the group consensus [24]. Any disagreements in coding were addressed through discussions with a third member of the research team.

## 3 Results

In total, 33 older consumers from Western Australia (n = 17) and Victoria (n = 16) participated in the forums. Briefly, 81.82% (n = 27) were aged between 65 and 84 years, 27.27% (n = 9) had been admitted to hospital four or more times in the past 5 years, and 6.06% (n = 2) spoke a primary language other than English at home; further details are presented in Table 1. Quotes were identified by table number (e.g. T2) and forum location (v for Victoria; w for Western Australia).

**Table 1:** Consumer characteristics.

Characteristics	WA consumers n = 17	VIC consumers n = 16	TOTAL n = 33
Age range			
45‐64	0 (0.00%)	1 (6.25%)	1 (3.03%)
65‐74	7 (41.18%)	5 (31.25%)	12 (36.36%)
75‐84	8 (47.06%)	7 (43.75%)	15 (45.46%)
85‐89	1 (5.88%)	3 (18.75%)	4 (12.12%)
90 or over	1 (5.88%)	0 (0.00%)	1 (3.03%)
Sex			
Male	6 (35.29%)	5 (31.25%)	11 (33.33%)
Female	11 (64.71%)	11 (68.75%)	22 (66.67%)
Primary language at home			
English	17 (100.00%)	14 (87.50%)	31 (93.94%)
Other^a^	0 (0.0%)	2 (12.50%)	2 (6.06%)
Current living arrangements			
Private dwelling alone	4 (23.53%)	7 (43.75%)	11 (33.33%)
Private dwelling with family	12 (70.59%)	8 (50.0%)	20 (60.61%)
Retirement village	1 (5.88%)	1 (6.25%)	2 (6.06%)
Capacity attending			
Older person (receiving care)	14 (82.35%)	11 (68.75%)	25 (75.76%)
Informal carer	3 (17.65%)	5 (31.25%)	8 (24.24%)
Number of hospital admissions in the past 5 years			
1	6 (35.29%)	6 (37.50%)	12 (36.37%)
2	3 (17.65%)	5 (31.25%)	8 (24.24%)
3	1 (5.88%)	3 (18.75%)	4 (12.12%)
4 or more	7 (41.18%)	2 (12.50%)	9 (27.27%)
Length of recent hospital admission^b^			
1 day	2 (11.77%)	5 (31.25%)	7 (21.21%)
2‐7 days	10 (58.81%)	6 (37.50%)	16 (48.49%)
8‐30 days	2 (11.77%)	5 (31.25%)	7 (21.21%)
> 30 days	2 (11.77%)	0 (0.00%)	2 (6.06%)
Reason for recent hospital admission			
Fall	1 (5.88%)	6 (37.50%)	7 (21.21%)
Respiratory	4 (23.53%)	1 (6.25%)	5 (15.15%)
Orthopaedic	1 (5.88%)	1 (6.25%)	2 (6.06%)
Neurological	1 (5.88%)	1 (6.25%)	2 (6.06%)
Cardiac	2 (11.77%)	0 (0.00%)	2 (6.06%)
Other^c^	8 (47.06%)	7 (43.75%)	15 (45.46%)
Fall during recent hospital admission			
Yes	0 (0.00%)	1 (6.25%)	1 (3.03%)
No	17 (100.00%)	15 (93.75%)	32 (96.97%)
Falls education received			
Yes	3 (17.65%)	1 (6.25%)	4 (12.12%)
No	14 (82.35%)	15 (93.75%)	29 (87.88%)

^a^Cantonese; Arabic, ^b^Missing data n = 1, ^c^Gastroenterology; Urology; Gynaecology; General surgery; Systemic infection.

Three generic categories describing effective implementation of the SRP at patient, ward and organisation levels emerged: (1) ‘tailor programme delivery' for each patient; (2) improve team communication to ‘keep everyone in the loop'; and (3) hospital leaders should own falls prevention, support training and facilitate information‐sharing to ‘join the dots' across departments and organisations. The group consensus was represented by the main category which was to ‘Make it [falls prevention patient education] a priority and work together'. Findings are described in detail below.

### 3.1 Tailor Programme Delivery for Each Patient

#### 3.1.1 Get the Delivery Time Right

There was strong agreement from participants across both forums regarding the timing of delivering the SRP. Participants felt the SRP resources should be provided shortly before or close to admission in a brief format to maximise information retention and avoid overload, T2w stated “*If you hit people with info when they first arrive to the ward, it's not going to stick*”. After admission, it was felt that patients should be allowed time to ‘settle in' especially if they were not well enough yet to process information, T3w commented “*Not too soon…*[patients] *may be too unwell to take in the information*” and T2v added “*When you have drugs in your system, you're not even aware*”. Once able, participants suggested the resources containing more detailed information could be provided as patients may have better capacity to focus and absorb the information. Another conversation point related to timing and patient safety, was the suggestion of pinpointing when falls were likely to occur and ensuring staff were available to check in on patients at these times.

#### 3.1.2 Make the Delivery Person‐Centred

Personalising and tailoring programme delivery for everyone was strongly recommended, with T1v emphasising it should be “*OUR falls prevention plan [patient‐centric] instead of Your plan* [generic]”, hence giving the patient ownership. Participants valued a one‐to‐one approach for receiving the education and commented that staff should work with the patient when developing their falls prevention plan, T1w “*Don't just barge into my room…talk to me, work with me*”. Some participants also highlighted the importance of providing a personal orientation to the correct use of equipment. For example, how to operate their call bell and change their bed height to ensure effective engagement with falls prevention strategies.

#### 3.1.3 Find Opportunities to Spread SRP Messages Using ‘The Right Places'

The placement of falls prevention resources was thought to be an important component in capturing patients' attention. Participants recognised the importance of placing relevant signage, electronic displays and posters in areas easy for patients to view. These included places within the patient's room (such as back of doors, bedside lockers or whiteboards on the wall), and communal hospital areas (such as the admission area, patient waiting rooms or hospital cafes). T2w noted “*Programme delivery needs to also consider the suitability of the environment ‘the right place'*, [as well as] *the right time*.” It was felt that signs and resources needed to have clear instructions and prompts (T1v), such as using phrases like “*has your loved one discussed their falls plan?*” to prompt meaningful discussion around their SRP plan. Another proposition was to have daily reminders of planned actions to prevent falls, T3w suggested to “*have Post‐it notes at the bedside to remind patients of their plan*”.

#### 3.1.4 Be Aware of Patients' Perspectives for Managing Risk

Participants pointed out that patients did not really see themselves as being at risk of falls particularly in a place of care, like a hospital, T3w “*Hospital is the last place you'd expect to fall*” and T1w “*You don't think about falling, especially in hospital, especially if it's an elective procedure*.” Therefore, the need to increase patients' awareness of their falls risk was raised, T3w “*We need more info about falls because patients are not normally thinking about falling*”. There was strong agreement that most patients were willing to take the risk of falling under certain conditions, namely to maintain their dignity such as when urgent toileting was required, T2w “*I would take the risk* [of falling] *over soiling the bed*”. It was identified that timely support by all staff, including consistent reinforcement of the same message throughout the day was required to mitigate patient engagement in risk‐taking behaviours, particularly in circumstances when patient dignity could be compromised, T2w “*It wouldn't hurt for the messages to be reminded to patients 2‐3x per day*”.

#### 3.1.5 Reach People From Culturally and Linguistically Diverse Backgrounds

A strong consensus arose from both forums about reaching people from culturally and linguistically diverse communities, T2w summed up “*Falls risks do not just apply to the old and Caucasian, many people are at risk for various reasons…the programme should appeal to the WHOLE community*.” Participants wanted resources to be able to reach this population and suggested that SRP text should be translated into relevant languages, subtitles to be included in videos, and that more visual aids should be utilised, T3v stated “*I know about 5 languages…* [translations needed in the northern area]”. The use of interpreters on the ward was also suggested for optimising communication in delivering the SRP.

### 3.2 Improve Team Communication – ‘Keep Everyone in the Loop'

#### 3.2.1 Clear Consistent Communication and Information Sharing Should Be Practised By Staff

Participants strongly identified that clear consistent communication should be practised on the ward amongst staff, as reflected by, T1w's experiences “*Communication between staff was disorganised*”. It was felt that staff needed to decide who would answer patient buzzers and communicate with different disciplines about falls risks for each patient. Another suggestion was to have different buzzers for different levels of patient urgency so that staff could prioritise actions, T1v *“I feel sorry for the staff… every time the buzzer is pressed, they have no idea what the person wants, there is no differentiation about relative needs* [urgency]… *someone might be dying”*. Participants also felt there should be a focus on patients' falls prevention plans during shift handovers, T2v “*they should discuss falls risk/management during handover and not just post fall*.” Handovers were also viewed as an opportunity for ‘checking in' and consistently reinforcing the patient's falls prevention plan, T1v *“A lot of the time staff don't know your history, they haven't read the report*…*there's a lack of communication.”*.

#### 3.2.2 Communicate With Family Members

Participants felt that family members were at times not provided with enough information or communication, T3w stated “*Family need regular phone calls from Doctors. These phone calls could include information on the patient's falls prevention plan*”. There was strong agreement that chosen family members should be kept informed at pre‐admission and during admission, and be provided the same falls prevention resources. Furthermore, they should be informed of who among ward staff to contact for information; T1v reflected “*My experience when family members visit…nurses don't know what is happening with their loved one”*. Participants suggested communication should occur through phone calls, emails and face‐to‐face discussions, as needed. Longer visiting hours and less visitor restrictions were also perceived as enablers for family and friends to provide more patient support around falls prevention.

#### 3.2.3 Family, Hospital Volunteers and Peers Can Be Safe Recovery Programme Supporters

When delivering the SRP on wards, participants suggested family members, could play an important role in supporting the patient, T1w reflected “*there's an unconscious sense of not seeing family as part of the team*”. However, some participants noted that it should be at the discretion of the patient as to which family members should be informed and involved, T1w “*Not all the family will be interested…need to filter who is included in conversations*. Other sources of support identified were hospital volunteers and peers. Participants suggested volunteers could give out programme information within their scope, T3w summed up “*Volunteers can speak to patients and explain falls prevention, while nurses look after patient care tasks*” thereby reducing staff burden. In one forum, a few participants reflected on their experience of how being in shared rooms could foster peer support amongst patients for engaging in their falls prevention plan, T3v “*Sometimes patients in shared rooms look out for each other*”.

#### 3.2.4 Ward Staff Need to Be Available and Accountable

Participants were aware of existing limitations around staffing numbers and caseloads, and a comment was made around how staff ratios needed to reflect patient ratios, T1v *“This person is run off their feet, this one is doing nothing”* which was acknowledged by many. Some participants suggested implementing staggered shift changes, so staff could be more available. There was also a need for accountability amongst staff and a change in ward culture. Participants strongly emphasised the need for team work on the ward, and that all staff needed to be informed and present during handover. The staff member leading falls prevention on the ward, a role hospitals often describe as ‘Falls champion', was viewed as highly important for conveying information to other staff, patients and family members. Participants reflected that the falls champion should be visible and easily identifiable as one participant recalled, T2v “*I always felt in the dark because I didn't know who to talk to* [about falls prevention]”. Wearing a lanyard or coloured sash were recommendations for improving identification.

### 3.3 Hospital Leaders Should Own, Endorse and Facilitate Srp Implementation ‐ ‘Join the Dots'

#### 3.3.1 Management Needs to Take Ownership of Falls Prevention Education

Participants in both forums agreed that for hospital falls prevention education programmes to be effective, hospital management needed to acknowledge their importance and take ownership of falls prevention education, starting at the executive level, T3w “*The CEO* [needs to] *acknowledge and raise awareness of the importance of the falls prevention education programme”*. Participants felt support from higher levels of hospital management would lead to increased confidence levels among both staff and patients regarding falls prevention.

#### 3.3.2 Endorsing Falls Prevention Education and Training for Staff and Volunteers

Participants strongly agreed that staff and volunteers needed to be supported through adequately funded falls prevention education and training, T3v “*Falls prevention workshops should be available to all volunteers and staff at least once a year, especially to new volunteers*”. Endorsement by hospital management of staff falls prevention education and training was also deemed vital in emphasising the scale of the problem of falls, T1,2v “*Train staff!* [training] *needs to have senior management provide them* [staff] *a reason for identifying it* [falls] *is a problem*.”

#### 3.3.3 Facilitate Inter‐Hospital Information Sharing for Broader Programme Implementation

**Figure 1:**
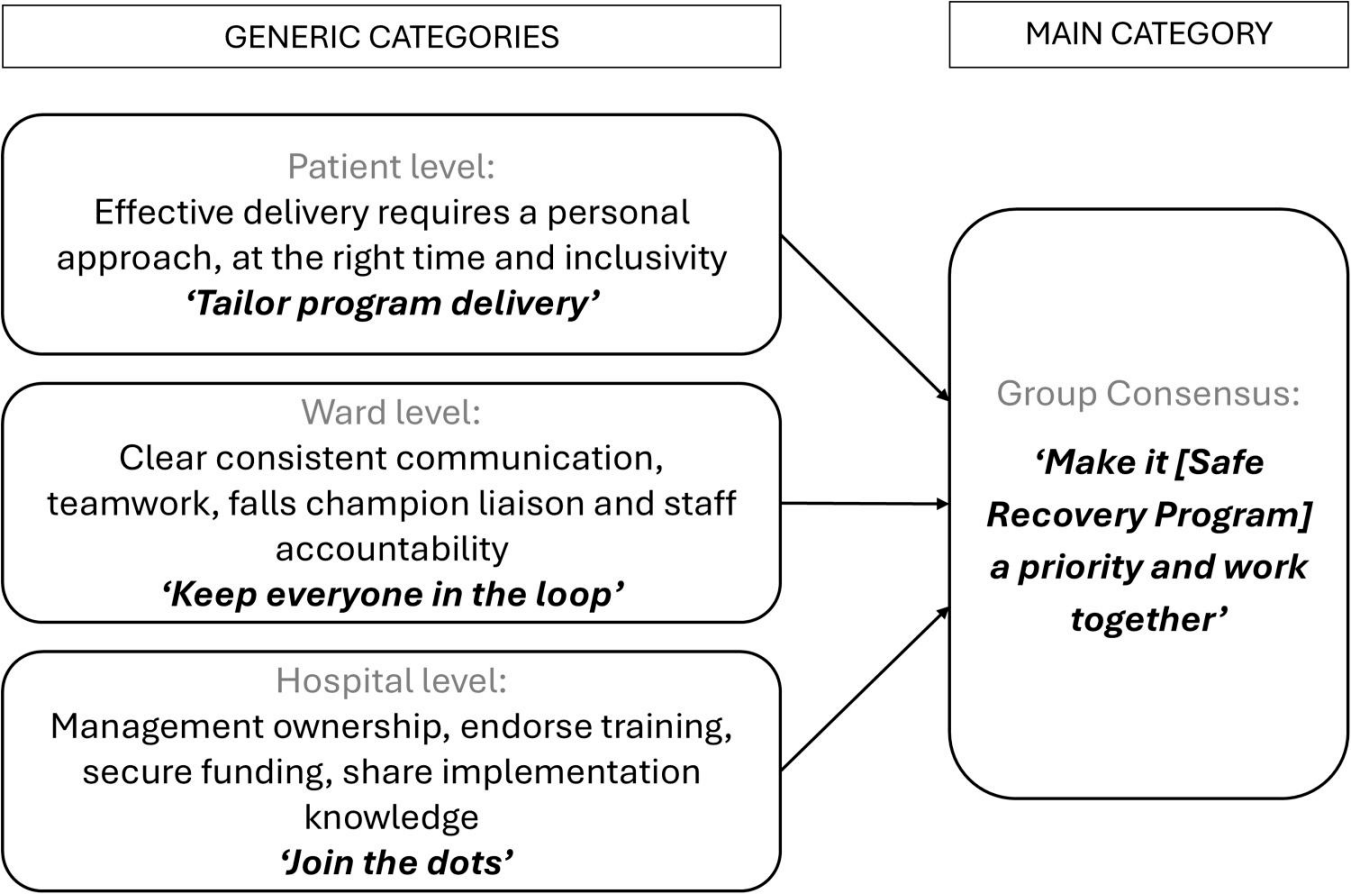
Final pathway to group consensus.

Both forums recognised a need for inter‐hospital information sharing to facilitate falls prevention from a broader perspective. Enabling the sharing of SRP resources, systems and patient records would allow for improved patient‐centred care and communication amongst different hospital staff, T2v “*Hospitals need to talk to each other* [through shared systems and records]”. This could ensure that falls prevention programmes or plans maintained continuity throughout a patient's health care journey (Figure 1).

### 3.4 Group Consensus – Make Patient Falls Prevention Education a Priority and Work Together

Overall, the forums concurred that management, staff and patients should make implementing falls education a priority and to do this effectively they all need to work together, “*There must be a whole of team approach*”. To effectively implement the SRP, consumers felt hospitals should focus on tailored and timely delivery, ensuring messages reach patients in the right places and at the right time. Delivery would ideally utilise a personalised approach, considering patients' differing perspectives about their risk of falls and engaging with patients from culturally and linguistically diverse communities. Collaboration and clear communication with families, hospital volunteers, and peers was recommended to consistently reinforce SRP messages. Utilising falls prevention champions, obtaining management ownership, and endorsing staff/volunteer training could strengthen programme adoption. Finally, inter‐hospital information sharing was deemed important for broader implementation and long‐term programme sustainability.

## 4 Discussion

Findings from this study provide meaningful new knowledge about older consumers' perspectives regarding what is needed to successfully implement falls prevention education on wards. Involving consumers in all stages of research, such as in this research preparing to deliver a new programme, increases the likelihood of uptake of health interventions and strengthens hospital safety, quality and outcomes for all stakeholders [20, 26, 27]. Participants' reflections about how falls education should be delivered are timely because older people have previously identified that staff do not raise their awareness or knowledge about hospital falls or educate them about how to reduce their falls risk in hospital [10, 11, 14, 28]. The group consensus was that the SRP was important and should be prioritised by everyone, with hospital leaders, ward staff, patients, family and volunteers working as a team for effective delivery. This consumer consensus is supported by other implementation research on hospital falls prevention, which identified that while implementation was a priority across multiple stakeholder groups, including patients, disempowerment and inability to understand who was responsible for falls prevention hindered effective implementation [29]. The literature also highlights challenges associated with implementing consumer recommendations due to the resource‐constrained nature of health systems, competing priorities on staff time, over reliance on just nurses being responsible for falls prevention and low‐value practices [30, 31, 32]. Workforce redesign utilising trained, supervised health assistants to deliver falls prevention education on hospital wards has been reported as one feasible solution in sharing responsibility at a clinical level [33].

Participants strongly suggested that falls education would be best received either prior to, or early in their admission when they were able to focus on the information and take appropriate preventative action. Findings strongly concur with a previous study where older people and caregivers with hospital experiences stated that implementing patient falls education could be enabled if delivery was timely and personalised for each patient [27]. The sparse number of randomised trials that have effectively reduced falls have used personalised education with timely staff delivery, confirming our participants' perspectives about what works for falls education [5, 12, 33].

Participants wanted to raise staff awareness that many patients had limited knowledge about falls and of the risk of falling whilst in hospital. They emphasised that staff should work with patients, as partners when developing their personalised falls prevention plan. Older patients have been shown to have very low levels of knowledge about falls in hospital and misjudge their self‐perceived risk of falling [10, 14]. A scoping review of hospital studies identified that two‐thirds of patients do not accurately identify their fall risk when compared to a health professional [34]. Engaging patients in tailored falls education has been shown to result in increased falls knowledge, risk awareness and a reduction in falls rates [15, 16, 17, 33].

Communication on hospital wards, both between staff and between patients and staff, was deemed a critical enabler for patient safety and a significant area for improvement, with the need to ‘*keep everyone in the loop*' strongly voiced by participants. Consumers have previously strongly expressed how poor communication about falls prevention meant they struggled to stay safe in hospital [14, 28]. Similarly older patients described how poor communication led to uncertainty and confusion about what actions they should take, like how to mobilise to the toilet in a timely manner while in hospital, as well as family members being uninformed [14, 28]. Communication challenges for CALD patients were perceived as an additional layer of complexity. The need for inclusive resources in a range of languages and the use of interpreters in hospitals to facilitate effective communication for CALD patients was highlighted in both forums, despite low CALD participant representation. Further research specifically involving CALD participants may provide stronger recommendations. It has been suggested that more than just language translation is required for the delivery of trustworthy, effective health education, culture must also be considered. Co‐design in negotiated partnerships with CALD communities could inform effective health education programmes and their delivery through relevant cultural lenses [35].

Regarding staff actions for falls prevention, participants reflected that ward culture needed to change with staff being more accountable for knowing patients' current falls prevention plan and being able to facilitate and communicate this information to other staff, patients and families. Participants agreed that the use of ‘falls champions' on the ward could facilitate improved communication and information sharing. However, this would require improved clarity regarding the leadership role of the falls champion, as others reported senior nurses were the ones making the important decisions regarding falls prevention on the ward [32]. Participants' reflections about accountability and communication, based on their hospital experiences, accorded with earlier research that we conducted where hospital staff provided feedback about implementing falls education. Staff suggested that barriers to delivering falls education included insufficient time for staff to deliver education and lack of falls prevention training for staff. Enablers suggested included sharing the responsibility to implement the programme, setting clear goals for staff, involving family to reinforce the messaging, and using falls champions to upskill staff [36]. Other research has also suggested hospitals could reconfigure ward‐based practices and develop electronic systems to better support this multidisciplinary and family involvement [30, 32]. The strong emphasis consumers placed on good communication and operating as a team is supported by the concept that falls in hospital are dependent on a three‐way interaction between staff, patients and the environment. Effective communication between staff and patients leads to mutual understanding, the actioning of falls prevention behaviours in a supported environment, and a reduction in falls [5, 29, 37, 38].

### 4.1 Strengths and Limitations

Having consumers share their experiences and ideas was a key strength of the study for ensuring authenticity in shaping decisions regarding implementation of the SRP. There were several limitations of this study. All participants needed to be able to travel to the research venue to participate in the world cafes; hence, some older people with moderate or severe motor or cognitive disabilities were unable to participate. Also, participants needed to be able to communicate in English; hence, people from culturally and linguistically diverse backgrounds were under‐represented in the sample. Only a small number of participants experienced a fall during a hospital admission or received falls education; thus, transferability and generalisability may be limited. We only collected data in Australia and future studies on falls prevention education in hospitals need to include a broader range of geographical settings, languages and cultures.

## 5 Conclusion

Older consumers provided valuable feedback to inform the implementation planning of a falls prevention education programme on hospital wards. Consumers concurred that teamwork between patients, staff and hospitals was a key implementation strategy that could foster effective programme delivery. Directly engaging with consumers potentially improves how falls education is delivered to patients in a meaningful and relevant way, which may contribute to more effective implementation during hospital admissions.

## Author Contributions

**Hazel Heng:** project administration, formal analysis, writing – original draft (lead), review and editing. **Jacqueline Francis‐Coad:** project administration, formal analysis (lead), writing – original draft, review and editing (lead). **Caroline Bulsara:** writing – review and editing. **Cheng Yen Loo:** project administration, formal analysis, writing – review and editing. **Meg E. Morris:** conceptualisation, project administration (co‐lead), writing – review and editing. **Adam Semciw:** project administration, writing – review and editing. **Catherine M. Said:** project administration, writing – review and editing. **Stephen Peterson:** conceptualisation (supporting), project administration (supporting), writing – review and editing. **Anne‐Marie Hill:** conceptualisation (lead), funding acquisition (lead), project administration (lead), formal analysis (co‐lead), writing – review and editing.

## Ethics Statement

Ethics approval for the study was obtained from the Human Research Ethics Committees of The University of Western Australia and La Trobe University (ID: 2024/ET000566). All participants provided written informed consent.

## Conflicts of Interest

The authors declare no conflicts of interest.

## Data Availability

The data that support the findings of this study are available from the corresponding author upon reasonable request.
